# Social behavior mediates the use of social and personal information in wild jays

**DOI:** 10.1038/s41598-022-06496-x

**Published:** 2022-02-15

**Authors:** Kelsey B. McCune, Jonathon J. Valente, Piotr G. Jablonski, Sang-im Lee, Renee R. Ha

**Affiliations:** 1 Psychology Department, University of Washington, Seattle, WA USA; 2 Department of Forest Engineering, Resources and Management, Oregon State University, Corvallis, OR USA; 3 Smithsonian Conservation Biology Institute, Migratory Bird Center, Washington, DC USA; 4 Laboratory of Behavioral Ecology and Evolution, School of Biological Sciences, Seoul National University, Seoul, South Korea; 5 Museum and Institute of Zoology, Polish Academy of Sciences, Warsaw, Poland; 6 Laboratory of Integrative Animal Ecology, Department of New Biology, Daegu-Gyeongbuk Institute of Science and Technology, Daegu, South Korea; 7 Present Address: Institute for Social, Behavioral and Economic Research, University of California, Santa Barbara, Santa Barbara, CA USA

**Keywords:** Behavioural ecology, Evolutionary ecology

## Abstract

The factors favoring the evolution of certain cognitive abilities in animals remain unclear. Social learning is a cognitive ability that reduces the cost of acquiring personal information and forms the foundation for cultural behavior. Theory predicts the evolutionary pressures to evolve social learning should be greater in more social species. However, research testing this theory has primarily occurred in captivity, where artificial environments can affect performance and yield conflicting results. We compared the use of social and personal information, and the social learning mechanisms used by wild, asocial California scrub-jays and social Mexican jays. We trained demonstrators to solve one door on a multi-door task, then measured the behavior of naïve conspecifics towards the task. If social learning occurs, observations of demonstrators will change the rate that naïve individuals interact with each door. We found both species socially learned, though personal information had a much greater effect on behavior in the asocial species while social information was more important for the social species. Additionally, both species used social information to avoid, rather than copy, conspecifics. Our findings demonstrate that while complex social group structures may be unnecessary for the evolution of social learning, it does affect the use of social versus personal information.

## Introduction

Studies of animal learning contribute greatly to our understanding of the function of cognition in natural systems^1^. The ability to learn from personal experience (also called asocial learning, trial-and-error learning, individual learning, or associative learning^2^) is likely evolutionarily conserved among bilateral animals^3^ and allows behavioral change in response to current information about the state of the local environment. Information about the environment produced through the actions of others (hereafter “social information”^4^) can also be used to change behavior to fit the current environment. The ability to change behavior after observation of, or interaction with, another animal or its products is called “social learning”^5^. Although it would seem that all species would benefit from social learning, researchers continue to find species that show no evidence for a social learning ability (i.e. mammals:^6^; birds:^7^). However, we have a limited understanding of when and why particular species exhibit social learning.

The prevailing hypothesis is that social learning should be more common in species that exist in larger groups^8,9^ where the frequency of encountering individuals producing information is higher than for relatively asocial species that exist in group sizes of 1–3^10^. By learning from observations of conspecifics, individuals may be able to quickly and efficiently adapt to their local environment^11^, and be more responsive to moderate environmental change^12,13^ in comparison with species that rely exclusively on personal learning or demonstrate primarily innate behaviors. For example, social learning is often required for mastering complex extractive foraging behaviors like tool use^14–16^, or the consumption of potentially harmful food like crabs and scorpions^17,18^.

On the other hand, a reliance on social learning can be maladaptive in some situations for both social and asocial species^19^. Social learning requires that individuals observe and remember conspecific behavior, which can inhibit the ability to acquire personal information about the current environment^10,20–22^. For example, Estrildid finches attending to informational cues from conspecifics about the quality of a foraging patch cannot also simultaneously acquire personal foraging information^23^. Therefore, asocial species, which inconsistently occur in groups, may lack the motivation or perceptual ability to attend to and remember the behavior of others when it results in a cost to personal foraging success^10,24^. However, it is unclear if individuals can choose between social and personal information if both are available and how that relates to their evolved social niche.

In addition to influencing whether individuals can, or choose to use social information, variation in social behavior could moderate *how* individuals socially learn. Social learning can occur through several cognitive mechanisms that govern which aspects of social information naïve individuals attend to and remember. This topic has been a focus for researchers interested in if and how human cognition and culture may be unique^25^. Researchers hypothesized that the different mechanisms require differing levels of cognitive demand^26^. The simplest mechanism, social facilitation, occurs when a naïve individual is more motivated to interact with, for example, a new food source, solely from the presence of conspecifics obtaining food there^25^. Additional learning mechanisms that are thought to be less cognitively demanding are stimulus and local enhancement, where a naïve individual uses social information about, respectively, a specific stimulus (i.e. a tool, a color) or location (i.e. hole in the ground, branch on a tree) to access a new food source^25,26^. These mechanisms can occur through associative learning where the actions of a conspecific become linked with a specific cue that predicts a reward^21^. In contrast, traditional studies of social learning in animals consider the mechanisms where the observer copies the behaviors of the demonstrator as the most cognitively demanding mechanisms because they are more likely to involve an understanding of another individual’s goals or intentions (“theory of mind”^27–29^). Copying mechanisms are divided into imitation, where many behaviors are precisely copied, and emulation, where the observer copies the demonstrator’s outcome but uses non-identical behaviors^26^. To distinguish among all of these social learning mechanisms, a task must require novel species actions and multiple methods for solving the task (i.e. a multi-access or two-action design^30,31^). However, experiments, especially those conducted on wild animals, often are not designed to distinguish among social learning mechanisms^25^.

Understanding the importance of social group structure on social learning requires testing individuals in natural groups in the wild. Otherwise, individuals with experience in laboratory-controlled social and physical environments may demonstrate unnatural behavior or artificially learned associations with social information that does not have relevance to the evolved species niche^32–35^. As an example, a study on an asocial species, the red-footed tortoise, found improved performance on a task after observation of a knowledgeable conspecific^36^. However, the tortoises in this study were housed together in captivity and therefore may have learned the relevance of social cues prior to the test^37^. As there is limited evidence for foraging-related social behavior in tortoises^38,39^, these results are difficult to generalize to understand social information use in the wild. To date, no studies have compared the ability to socially learn and social learning mechanisms in wild species that differ in social behavior^11,40–42^.

Here we conducted a social learning experiment in the wild, with individually marked birds. We chose two congeneric species that differ in social behavior, the California scrub-jay (hereafter “CASJ”; *Aphelocoma californica*) and the Mexican jay (hereafter “MEJA”; *A. wollweberi*). No avian species is exclusively asocial^43^, but we chose CASJ because they exhibit a pair-bonded monogamous breeding system and aggressively defend their territory from other jays, including their own offspring that have become independent^44^. This leads to a narrow window in which the young can learn from parents, and no consistent opportunities for adults to take advantage of knowledge acquired by conspecifics. In contrast, MEJA are communal, cooperative breeders where young delay dispersal, leading to stable groups of 5–25 jays^45^. Therefore, MEJA have a consistent social environment in which they can observe and learn about foraging opportunities from group-mates throughout life^8^. Even though the social behavior is distinct, these two species occupy similar ecological niches; both depend on tree nuts such as acorns and are opportunistic generalist foragers that prefer dry, open, scrub-oak and pine habitats^46^, and cache food items at similar rates^47^. Consequently, any differences in the expression of a social learning ability or social learning mechanism are most likely the result of divergent pressures from the social environment, and not differences in the foraging niche.

**Figure 1:**
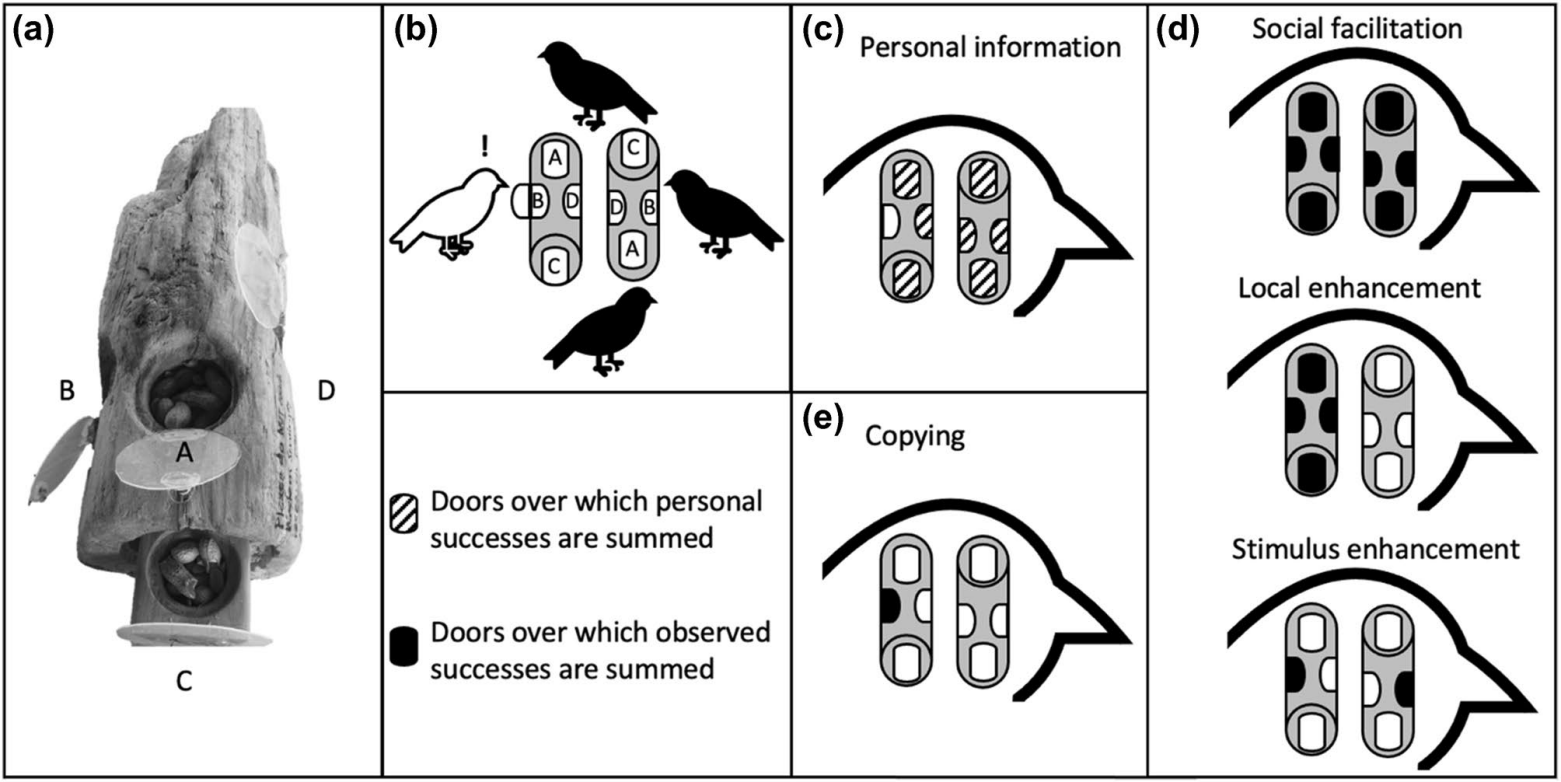
(**a**) Our social learning experiment involved placing food behind each of four door types on two identical apparatuses created out of logs. Doors with the same letter are the same door type and use identical locking mechanisms. (**b**) This resulted in 8 potential doors that each naïve bird (white) could attempt to open (here B door on the left apparatus). Attempts at a specific door can be motivated by (**c**) personal information from opening any of the other 7 doors, or (**d**–**e**) social information from observing conspecifics open any of the 8 doors. The number of observed successes at different combinations of door types and apparatus locations (left or right) are used as covariates to test for evidence of (**d**) simpler, or (**e**) the more cognitively demanding social learning mechanisms of imitation and emulation (Table 1).

We trained “demonstrator” jays from each species on a method for opening one door on a multi-door foraging task (Fig. 1), but we used an open-diffusion design so any individual that innovates a method for opening a door can serve as an additional demonstrator. We presented the foraging task to natural groups composed of demonstrators and naïve birds in their local environment, where we define a bird as “naïve” to a specific door on the multi-door apparatus if it has never personally attempted to open that specific door. We tested whether the rate at which naïve birds interact with each novel door on the multi-door task is increased or decreased by personal experience solving other doors on the apparatus (personal information) or observations of conspecifics solving doors (social information). If more complex social group structures favor the use of social learning, we predicted that the rate at which naïve MEJA (social species) interact with novel doors would be strongly affected by the number of observed successes of conspecifics. We predicted that the more asocial CASJ, on the other hand, would not attend to social information and instead would rely on the memory of their previous successes (personal information) to guide where they subsequently interact with the task. However, if both species use social information, we predicted that the more social species will use the copying mechanisms of emulation or imitation while the asocial species will use cognitively simpler social learning mechanisms like social facilitation, stimulus, or local enhancement (Table 1, Fig. 1).

## Methods

### Study system

Subjects for this experiment included 49 individually color-banded MEJA from five flocks around the Southwestern Research Station in Portal, AZ and 26 individually color-banded CASJ from 16 territories in Willamette Mission State Park in Keizer, OR. To measure the transmission of social information, our study required aggregations of naïve individuals with demonstrators knowledgeable about how to solve a novel foraging task. MEJA live in natural stable groups (flocks), and social learning trials were conducted on each of the five groups’ territories. For the more asocial CASJ, we created five groups from a minimum of three neighboring territories surrounding central, open picnic areas that we determined were not defended by any of the jay pairs. Trials conducted in these “neutral zones” mimic natural situations where dispersing hatch-year and adult CASJ interact at large, seasonal masting trees in the fall^48^. All jays were trained to come for peanuts when we whistled, which facilitated timely participation in the social learning task. All methods described here were approved by the University of Washington Institutional Animal Care and Use Committee (protocol #4064-03), the Southwestern Research Station, and the appropriate permitting agencies (Bird Banding Lab #22802, Oregon Department of Fish and Wildlife #015-16, Oregon State Parks #007-14, US Fish and Wildlife Service #MB51894B-0, Arizona Game and Fish Department #SP697293). We followed all guidelines for ethical animal research outlined in the Association for the Study of Animal Behaviour Guidelines for the Treatment of Animals in Behavioural Research and Teaching.

### Experimental apparatus and habituation

We created the apparatuses for our multi-door foraging task from two identical logs to mimic natural extractive foraging or cache-recovery behaviors^45^. Each apparatus had four food-holding compartments covered by transparent plastic that opened in different ways (hereafter “door types”; Fig. 1a). We added simple locks to three of the four door types so that they could only be opened with novel behaviors which increased the difficulty of the task to facilitate detection of social learning^25^. Locking mechanisms were identical between analogous door types on the two apparatuses. The fourth door type on each apparatus was left unlocked and filled with a less preferred food item (sunflower seeds rather than peanuts); this prevented naïve jays from abandoning the foraging area due to failure to obtain food, thereby increasing their probability of observing groupmates interacting with the task. We habituated jays to the puzzle boxes (described in the electronic supplementary material S1) until they were eating from the compartments quickly and without behaviors indicative of fear (jumping away) before starting experimental trials.

**Table 1:** Definitions for the learning mechanisms examined in this study, and the locations in Fig. 1 where the methods for measuring each mechanism are illustrated.

Mechanism	Definition	Methods
Personal information	Animals adjust their behavior to the local environment through individual experience, leading to associative learning^2^	Figure 1c
Social facilitation	The mere presence of conspecifics obtaining food increases the probability of naïve individuals interacting with the task^25^	Figure 1d
Local enhancement	A demonstrator obtaining food from a particular location, leads naïve individuals to be more likely to visit or interact with objects at that location^25^	Figure 1d
Stimulus enhancement	The activity of a demonstrator draws the attention of naïve individuals to a particular type of object^49^	Figure 1d
Copying	After observing a demonstrator interact with objects in the environment, naïve individuals are more likely to use similar behaviors to interact with the same task to achieve a similar outcome (imitation or emulation^25^)	Figure 1e

### Demonstrator training

To seed knowledge of the lock-opening methods into the groups, we trained two adult demonstrators from each social group^50^. However, as our experiment took place in the natural group setting, any jay that innovated a solution to any door during trials served as a demonstrator. Due to the differences in social systems, we used slightly different methods between species to isolate jays for training as demonstrators. We trained CASJ in the center of their territory where the aggressive territoriality meant that neighboring jays could not move close enough to observe. For social MEJA it was impossible to separate two individuals from groupmates for training in the wild. Therefore, we took two MEJA from each of the 5 flocks temporarily into captivity in large aviaries on the campus of the Southwestern Research Station. We trained a male and female CASJ mate pair as demonstrators, but we could not balance the sex ratio of MEJA demonstrators because it is impossible to discern the sex morphologically or behaviorally during the non-breeding season. For both species, the individuals we chose to train as demonstrators were adults (> 2 years old) and the most dominant individuals in each group (dominance assay methods as in:^51^). The methods to train demonstrators to open one of the locks were identical between species and are described in detail in S1.

### Data collection

The multi-door foraging apparatuses were designed to not only determine *whether* jays socially learn, but also *how* individuals socially learn by distinguishing among mutually exclusive social learning mechanisms (imitation, emulation, local enhancement, stimulus enhancement, or social facilitation). We initially planned to test for imitation, but after training, the demonstrator jays did not consistently use the specific action on which they were trained to open the lock (e.g., they would swivel the lock both up and down rather than in one consistent direction). Consequently, naive jays that opened the same door on the same log as the demonstrators were best described as using ‘end-state emulation’^26^, and we refer to this behavior as ‘emulation’ hereafter. On each trial we presented the two identical apparatuses 2 m apart and facing alternate directions; this, along with the 4 separate door types, allowed us to disentangle which social learning mechanism jays use (Table 1, Fig. 1).

For all groups, we conducted between 9 and 16 trials in the non-breeding season that were each a maximum of 2 h in length. The number of trials varied due to differences in the duration of breeding behavior in each social group and logistical time constraints. Trials were conducted in the mornings before noon, and only one trial was conducted per day per group. To begin a trial, we deployed the two apparatuses in the center of each group area with all doors unlocked, open and baited with a food item. We determined that jays were motivated to engage with the task if members of the social group arrived and ate within 10 min. If no jays arrived, the trial attempt was aborted. If at least one subject (excluding trained demonstrators) arrived and ate within 10 min, we refilled compartments, closed, and locked all doors and began the trial.

All trials were filmed, and behaviors coded later by research assistants naïve to our hypotheses (see S2 for full ethogram and coding details). We considered a jay to have interacted with a door or lock when they contacted it with a foot or bill. Each time a jay interacted, we recorded: the amount of elapsed trial time, the individual’s identity, the apparatus location (left or right) and door type (A, B, C or D; Fig. 1), whether a food item was successfully obtained, and the identities of all other individuals within 10 m that likely observed the interaction. Scrounging events, where a jay obtained a food item by stealing it from the jay that opened the door (Table S2.1), were tallied separately. With these data we can quantify each individual’s personal experiences opening each door type at each apparatus location, and the opportunities to socially learn door-opening methods via the 4 social learning mechanisms (Fig. 1).

### Data analysis

First, we ensured that sampling effort was equivalent between the two species by comparing the number of trials, amount of trial time, the number of participating jays, the number of demonstrations and the number of times jays observed demonstrations. We used non-parametric analyses (Mann–Whitney U Test) because these count variables were all either over- or under-dispersed.

We used mixed effects Cox proportional hazards models (Cox PH) to examine whether the probability a jay first interacts with a novel door at a given moment in time (hereafter, interaction rate) changes as a function of their own prior successes (evidence for personal learning) and/or their observations of other jays succeeding (evidence for social learning; Fig. 1). Cox PH is a type of survival model used to link the time it takes for an event to occur with covariates that could affect the rate at which they occur. This is appropriate for our social learning data because we have information about the time it took for each jay to first interact with each door type at each apparatus location, in conjunction with the number of times they previously observed conspecifics solving that door and the number of times they personally solved other doors. This is a statistically rigorous approach for evaluating learning^25,52–54^ and has several advantages. First, Cox PH models allow explicit analyses of covariates that change with time (hereafter time-varying covariates^55^). Both of our covariates of interest (the number of personal successes a jay has, and the number it observes) increase throughout the study for each individual, potentially altering the bird’s interaction rate with new doors; using a Cox PH model ensures the bird’s knowledge can be updated with each new experience. In addition, survival models are designed to handle right-censored data which, in our study, occurred when a bird never visited a particular door before all trials concluded. Using Cox PH models allowed us to retain those observations which can contribute to our understanding of how personal and observed experiences influence behavior.

We purposefully chose time to first interaction at each door as our response behavior rather than time to first success. The rate at which each door type is first *attempted* is more directly attributable to social information compared to the time to first solve^52,56^. Due to the complexity of the door locks, very few jays were successful on the first interaction with a novel door type and any time elapsing between the first interaction and first solve is influenced by individual characteristics of persistence and motivation, rather than social information^35^. The effect of observed experiences on the amount of time elapsed before the first interaction is thus a better test of whether jays use social information to initially approach a novel challenge.

For each species, we constructed a baseline Cox PH model (see S3 for model specifications) where interaction rate was a function of age (adult or juvenile), habituation time (amount of time the individual was exposed to the unlocked, open apparatus prior to the experiment), and a time-varying covariate representing scrounging events (the number of times the individual was able to steal food from any compartment or conspecific without opening the door during the experiment). Each individual was initially naïve to how to open either six (demonstrators) or eight doors (four door types on two spatially separate apparatuses) and therefore could have six or eight potential first interactions; thus, we included a random effect of individual identity to account for non-independence of these observations. We also included random intercepts controlling for correlations among individuals from the same group, doors on the same apparatus and identical door types across the two apparatuses. Because this baseline model included no personal or social learning mechanism covariates, it represents the null hypothesis that first interactions with each novel door are random with respect to personal or observed experiences.

To test for the influence of each learning mechanism we created 5 unique time-varying covariates (Table 1, Fig. 1). The personal information covariate was quantified as the number of times the individual had acquired food at any of the seven other doors prior to first interacting at the novel focal door. We quantified the social facilitation covariate as the number of observed successes at any of the eight doors, the local enhancement covariate as the number of observed successes on any of the four doors (regardless of door type) on the specific apparatus where the novel focal door was located, stimulus enhancement as the number of observed successes at the specific door type (at either of the two apparatus locations), and emulation as observations of successes at that exact location and type of the novel focal door prior to first interacting there.

For each species, we incorporated an additive effect of each of the five learning covariates into the baseline model individually to test the hypothesis that individuals only learn via personal information or one social learning mechanism. To test the hypothesis that birds learn via both personal and social information, we additionally built on the personal learning model by including an additive effect of each social learning mechanism individually. Finally, we tested the hypothesis that the effect of social information changes as personal information is acquired by also including an interaction between personal information and each social learning mechanism. We did not include more than one social learning mechanism in any model because they tended to be correlated with one another (r > 0.5) and because we were interested in distinguishing among these mechanisms in terms of how each species learns. All models were fit using the coxme package^57^ in R version 3.5.1.

This resulted in 14 models for each species that we compared using Akaike’s Information Criterion (AIC^58^) based on the penalized likelihood. As noted, social learning mechanisms tended to be correlated with one another which could result in several models having strong support (i.e., within two AIC units of the top model^59^). Thus, we calculated the relative importance of each learning mechanism by summing the AIC weights of all models in which the variable was included. This approach is used to determine how important a predictor is relative to others being considered^59^. Note that the personal information covariate was included in nine models, but each social learning mechanism was only included in three. Consequently, we only summed the weights over the top three models in which personal information was included to ensure that relative importance was comparable^59^. Finally, we tested the proportional hazards assumption of our top models to ensure they provided a reasonable fit by visually and statistically testing for an effect of time on scaled Schoenfeld residuals^55^.

## Results

Jays from both species had equal opportunity to interact with and observe conspecific interactions at the multi-door foraging apparatuses. There was no significant difference in the number of times that a MEJA or CASJ demonstrator opened the door type that it was trained on (CASJ mean ± standard error = 110.2 ± 33.3, MEJA = 75 ± 36.0; *W* = 16, *p* = 0.53), or in the number of times that naïve individuals of each species observed a demonstrator open a door type (CASJ = 8.68 ± 1.6, MEJA = 10.04 ± 1.6; *W* = 229.5, *p* = 0.60). There were also no differences between species in the number of trials (CASJ = 13.2 ± 0.8, MEJA = 13.2 ± 1.2; *W* = 13, *p* = 1), total minutes of trial time that each social learning group experienced (CASJ = 1146 ± 75.6, MEJA = 1235.8 ± 141.2; *W* = 9, *p* = 0.55), or in the average number of jays in each social learning group (CASJ = 7.4 ± 0.9, MEJA = 9.6 ± 0.8; *W* = 5, *p* = 0.13). MEJA did, however, tend to attempt on more doors; the mean percentage of doors attempted across MEJA individuals was 65.2% (SE = 4.9) and only 54.6% (SE = 6.1) for CASJ (Fig. S4.1).

**Figure 2:**
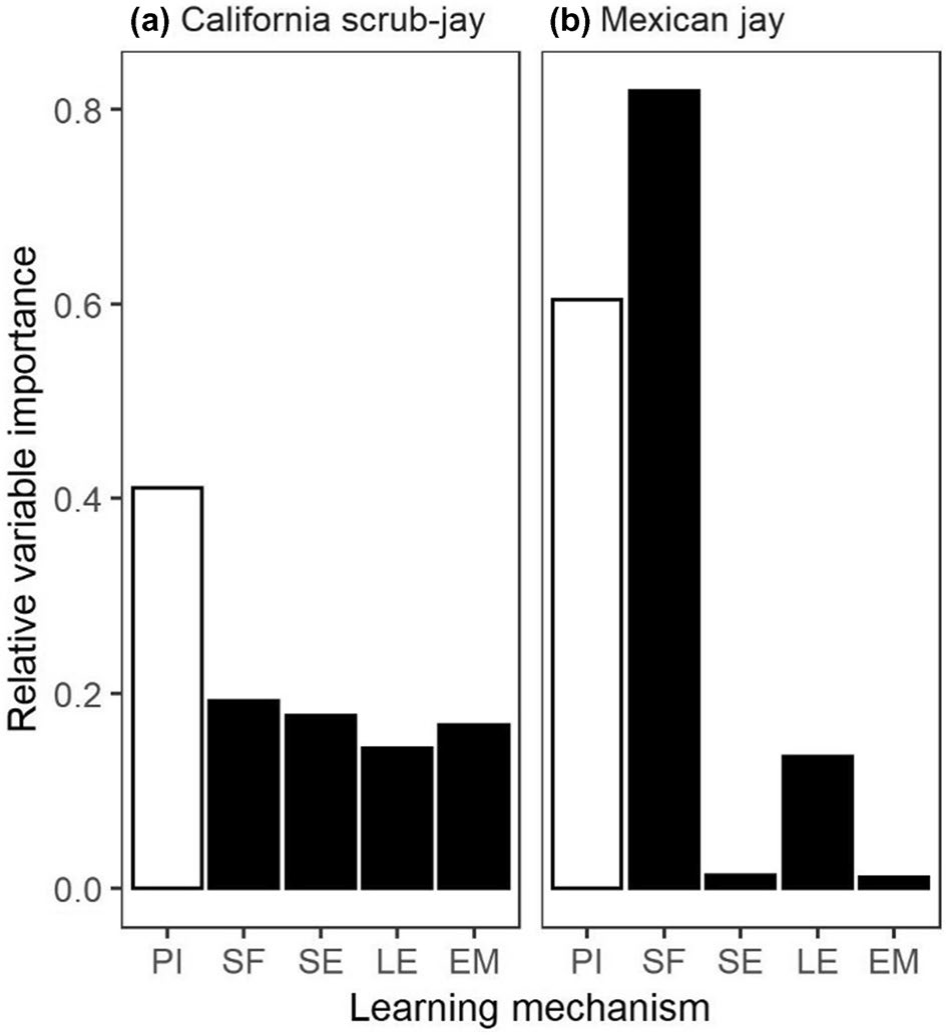
The relative importance of the variables representing personal information (in white; PI) and four social learning mechanisms (in black; social facilitation [SF], stimulus enhancement [SE], local enhancement [LE] and emulation [EM]) on the rate of first interaction with novel doors on the foraging task for each species. Values were calculated by summing the AIC weights from the top 3 models in which each variable occurred (Table 2).

**Figure 3:**
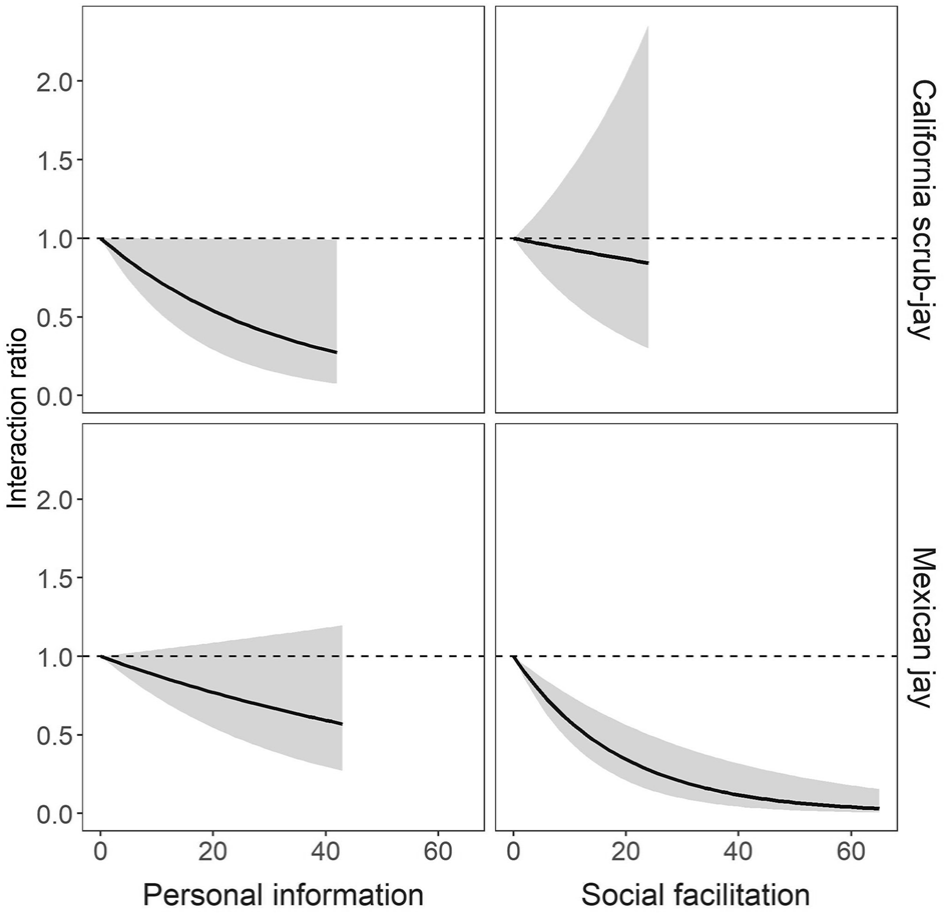
Top models for both the California scrub-jay and Mexican jay indicated that the probability of interacting with a novel door on the foraging task decreased as the individual gained information (personal foraging successes on other doors and observed foraging successes on all doors). The y-axis shows interaction ratios predicted from these top models which describe the instantaneous probability of an individual interacting with a novel door relative to a naïve bird with no personal or social information. The dashed line at y = 1 indicates the focal bird is equally likely to interact at a novel door as the naïve bird.

Results from our model comparison indicate that while asocial CASJ primarily used personal information to guide their interactions with the novel doors on the foraging task, social information can influence their behavior as well. Six models had some support (i.e., < 2 AIC units from the top model), including the baseline model and models containing all five learning covariates (Table 2). That said, the top CASJ model, which had more than 2 times as much support as any other model, included personal information, but no social learning mechanism covariates. When assessing relative importance of each covariate, personal information was over twice as important as any of the social learning mechanism covariates (Fig. 2). Personal information had a negative effect on CASJ interaction rates; the top model indicated that the relative probability of the focal individual attempting at a novel door decreased by 3% (95% CI = [− 9%, 3%]) with each personal success at known doors (Fig. 3). Our test of model fit indicated no evidence that any covariates in this top model violated the proportional hazards assumption (*χ*^*2*^ = 12.20, df = 34.8, *p* > 0.99). The top social learning mechanism (social facilitation) was also negatively associated with interaction rate as the relative likelihood of attempting a novel door decreased by 1% (95% CI = [− 9%, 8%]) with each observed success of conspecifics at any door.

**Table 2:** AIC comparison of models evaluating the effects of personal information and four social learning mechanisms (social facilitation, stimulus enhancement, local enhancement, and emulation) on the rate at which two jay species interact with novel doors on a multi-door foraging task. All models built on the baseline model which included random effects for group, individual, door type, and apparatus location, as well as fixed effects for individual age, habituation to the puzzle box, and previous success scrounging.

Species	Model	DF	Log likelihood	AIC	Delta AIC	AIC weight
California scrub-jay	Personal	26.81	− 570.54	1194.70	0.00	0.22
	SocialFacilitation + Personal	27.71	− 570.41	1196.23	1.53	0.10
	Baseline	25.41	− 572.72	1196.26	1.56	0.10
	StimulusEnhancement + Personal	27.81	− 570.44	1196.50	1.80	0.09
	LocalEnhancement + Personal	27.60	− 570.74	1196.67	1.97	0.08
	Emulation + Personal	27.71	− 570.63	1196.68	1.98	0.08
	SocialFacilitation	26.43	− 572.27	1197.39	2.69	0.06
	Emulation*Personal	28.66	− 570.19	1197.71	3.01	0.05
	StimulusEnhancement	26.46	− 572.48	1197.89	3.19	0.04
	StimulusEnhancement*Personal	28.73	− 570.25	1197.96	3.26	0.04
	Emulation	26.33	− 572.79	1198.25	3.55	0.04
	LocalEnhancement	26.18	− 573.01	1198.37	3.67	0.03
	SocialFacilitation*Personal	28.48	− 570.77	1198.51	3.81	0.03
	LocalEnhancement*Personal	28.33	− 571.14	1198.93	4.23	0.03
Mexican jay	SocialFacilitation + Personal	46.85	− 1152.54	2398.78	0.00	0.39
	SocialFacilitation	45.77	− 1153.91	2399.37	0.59	0.29
	SocialFacilitation*Personal	47.72	− 1152.75	2400.95	2.17	0.13
	LocalEnhancement + Personal	46.60	− 1154.40	2402.00	3.22	0.08
	LocalEnhancement	45.44	− 1156.34	2403.56	4.77	0.04
	LocalEnhancement*Personal	47.46	− 1154.90	2404.71	5.93	0.02
	Personal	45.61	− 1156.88	2404.97	6.19	0.02
	StimulusEnhancement + Personal	46.61	− 1156.77	2406.76	7.98	0.01
	Emulation + Personal	46.61	− 1156.84	2406.89	8.10	0.01
	StimulusEnhancement*Personal	47.72	− 1156.21	2407.84	9.06	0.00
	Baseline	44.41	− 1159.58	2407.98	9.20	0.00
	Emulation*Personal	47.57	− 1156.92	2408.97	10.19	0.00
	StimulusEnhancement	45.45	− 1159.29	2409.47	10.69	0.00
	Emulation	45.43	− 1159.40	2409.64	10.86	0.00

On the other hand, we found strong evidence that social MEJA use both personal and social information to guide their interactions with novel doors on the foraging task. The top model contained covariates for personal information and social facilitation, while the only other model with substantial support contained social facilitation and excluded personal information (Table 2). Thus, although we hypothesized that this species would use a more cognitively demanding social learning mechanism like emulation, social facilitation was the most important learning mechanism covariate, and was more important than personal information (Fig. 2). As with CASJ, personal information and social facilitation negatively influenced interaction rate. The top MEJA model indicated a 1% decline (95% CI = [− 5%, 2%]) in the relative probability of attempting a novel door with each personal success at known doors, and a 5% decline (95% CI = [− 10%, 0%]) with each observed success by a conspecific (Fig. 3). Again, we found no evidence that any covariates in this top model violated the proportional hazards assumption (*χ*^*2*^ = 3.72, df = 55.9, *p* > 0.99), suggesting it was a reasonable fit for the data.

## Discussion

We found support for the hypothesis that sociality drives the use of social information. Although the behavior of individuals from the social species (MEJA) towards novel doors on our multi-door foraging task was strongly affected by both social facilitation and personal information, social facilitation was much more important. That said, MEJA did not rely on the more cognitively complex social learning mechanism of emulation as we predicted. While there was some evidence that asocial CASJ can use social information, interactions with novel doors were much more strongly influenced by personal information than any of the social learning mechanisms. We note that the baseline model did have some support, which would indicate that CASJ may interact randomly because they are able to see food behind all doors. That said, summed AIC weights indicate there is a < 10% chance this is the true model underlying the data, and consequently a > 90% chance that the correct model includes at least one learning covariate. Interestingly, we found that, for both species, increased social information resulted in naïve individuals avoiding doors where conspecifics succeeded. This is unlikely to be an artifact of dominant demonstrator jays blocking others from accessing the puzzle box because jays typically leave the area to cache the peanut after opening a door, allowing other individuals the option to approach the compartment. Collectively, this suggests that individuals were using social information to choose to avoid competition rather than to exploit novel foraging opportunities discovered by conspecifics.

Despite a primary reliance on personal information, our finding that asocial CASJ behavior can be influenced by social information is in-line with previous research on CASJ cognition. Clayton and colleagues^60^ found that CASJ tested in captivity showed evidence for “theory of mind” (the ability to infer the knowledge state of others) when protecting the location of cached food from competing jays. Theory of mind is a cognitive ability that is potentially important for navigating social interactions and has previously only been thoroughly documented in great apes^61^. That the relatively asocial CASJ possesses the ability to use theory of mind and social learning indicates that there are potentially more nuanced aspects of social interactions affecting the evolution or development of cognitive traits than can be captured by measures of group size and breeding system alone^62^. For example, although CASJ form monogamous pair bonds after sexual maturity, juveniles in some populations temporarily join non-breeding flocks^63^ where early learning of the relevance of social cues could occur (i.e. common ravens^64,65^). We did not, however, see evidence of non-breeder flocks in our CASJ population in Oregon, despite banding a considerable number of juveniles.

As the common ancestor of species in the *Aphelocoma* genus was likely social, it is possible that cognitive abilities like social learning were not lost when CASJ lost sociality^66–69^. In fact, species in the *Aphelocoma* genus notably exhibit wide variation in social behavior, indicating that it is a fairly plastic trait^70^. The most supported phylogenetic tree indicates the common ancestor of *Aphelocoma* species was likely a singular cooperative breeder^68,69^ with a breeding system similar to that of the extant Florida scrub-jay (*A. coerulescens*) where young delay dispersal to help their parents raise siblings in subsequent breeding attempts. Research on juvenile Florida scrub-jays revealed they possess the ability to socially learn from parents about foraging opportunities in a novel patch^71^. Social learning from parents during development (vertical transmission of information) can result from genetically determined juvenile-specific processes or innate behaviors (e.g. bird song learning, imprinting^72^) that may not be analogous to social learning among adults (horizontal transmission of information). Nevertheless, it is possible that CASJ can use social information due to retention over evolutionary time from a more social ancestor and future research should test the ability of Florida scrub-jays and other *Aphelocoma* species to socially learn via horizontal transmission.

We found strong support that both studied species use social facilitation, where the mere presence of a conspecific at the task affects the behavior of naïve individuals. MEJA have consistent, close interactions with groupmates throughout life, so naïve individuals have greater exposure to a variety of social information. Thus, we predicted that MEJA might use the more cognitively demanding social learning mechanisms to discriminate the relevant social information for a novel foraging task^73^. Furthermore, in cooperative species, high levels of tolerance and affiliative behaviors may enable naïve individuals to be closer to knowledgeable groupmates so that the resolution of the novel foraging behavior is higher^74^. However, it is possible that the natural environment of *Aphelocoma* jays does not contain extractive foraging problems that are complex enough to warrant cognitively demanding mechanisms like emulation. Yet, there is some evidence that social facilitation also requires understanding another’s goals and intentions, components of theory of mind^73^. As such, more research on the underlying cognitive processes required for each social learning mechanism is needed to clarify similarities in the learning processes of humans and other vertebrate species.

In contrast to many studies on social learning^25^, we found that social information had a negative, or dampening, effect on interactions with novel doors for both species. Although avoidance of conspecifics is necessary for the cache-protection strategies of CASJ, this is more surprising for MEJA. As a social and cooperative species, the exploitation of social information might be expected to result in individuals being *more* likely to use new food sources^75^. However, while access to up-to-date social information is a benefit to living in a social group, competition for resources can be a major cost^76^. For example, chimpanzees also use social information to out-compete and avoid conflict with groupmates^77,78^. Our results imply that both CASJ and MEJA use social information to minimize potential competition with conspecifics at novel foraging locations and instead continue to acquire food from familiar locations with known risks.

The functional impact of social learning on survival and reproduction depends not on whether species have the capacity for an ability, but when and how they use it in their natural environment. To increase the validity and repeatability of experiments testing the relationship between sociality and social learning, it is important for future research to extend experiments outside of the laboratory. The captive environment alters social group dynamics and cognitive abilities in various ways compared to wild conspecifics, preventing the generalization of results from studies on captive individuals to the species as a whole^35^. For example, captive keas were able to use social learning to solve a novel foraging task^79^, but wild keas failed to show evidence for social learning^80^. It is extremely difficult to simulate natural group dynamics and diffusion of information in captivity^81,82^, and some species may not socially learn in the absence of natural social interactions^83^. Cognitive assessments in the natural environment, where social interactions and individual movement patterns are unrestricted, are often feasible (as demonstrated here) and will result in more interpretable and ecologically relevant performance^35,84,85^.

While it seems intuitive that the ability to socially learn is most likely to evolve in social species, very few studies have explicitly tested this. We know of no other studies evaluating the relationship between social group structure and social learning performance by comparing wild species that differ in social behavior on an experimental task, though a few studies have tested this in captivity^86,87^. Elucidating the factors affecting the use of personal and social information of wild individuals can facilitate conservation management for threatened species^13,88–90^, increase our ability to predict the impact of environmental change on animal populations^91^, and help to better understand the evolution of human cognition^92,93^. The research presented here is therefore an important step forward in our understanding of the influence of complex social group structure on this aspect of cognition.

## Supplementary Information

Supplementary Information.
