# Supplementary material for: Social behavior mediates the use of social and personal information in wild jays
Source: Sci Rep. 2022 Feb 15;12:2494. doi: 10.1038/s41598-022-06496-x (PMC8847367; doi:10.1038/s41598-022-06496-x)
Supplement: Supplementary file 1 — Supplementary Information. [file 41598_2022_6496_MOESM1_ESM.docx]

**Supplementary information**

S1. Supplemental methodological details

All jays were trapped using baited walk-in traps or mist nets. After capture we applied unique combinations of colored plastic leg bands and an aluminum USGS numbered band, took a blood sample and released the jay at the location of capture.

Prior to our social learning experiments, we trained jays to recognize the apparatus as a food source. First, in the center of the territory of each of five MEJA flocks or five CASJ neutral zones we allowed the group access to one open apparatus filled with peanuts until they habituated to this new food source. Second, we standardized jay knowledge of the simple door opening methods by giving all jays experience interacting with the closed (but unlocked) apparatus doors (see methods in McCune et al. 2019). After habituation to the simple apparatus doors, we added unique locks to three of the four doors (Figure S1.1). To encourage attendance of naïve jays at the apparatus during learning trials (thereby increasing their probability of observing groupmates), we left the right-side door unlocked, but filled the compartment with a less preferred food (sunflower seeds rather than peanuts).


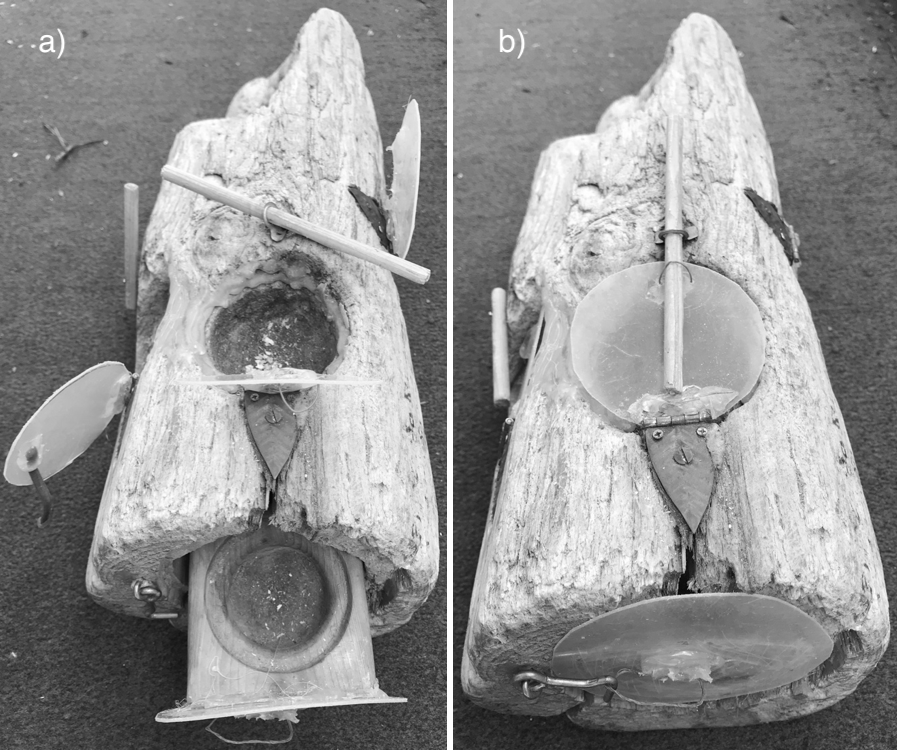
Figure S1.1: The puzzle box apparatus with a) doors open and unlocked, while b) shows the doors closed and locked. There are locks on 3 of the 4 doors that open in different ways: The stick lock on top can be pushed or pulled out of the loops, the stick lock on the left can be swiveled up or down across the door, while the lock on the front is a hook-and-eye style lock that must be lifted up to open the door.

We trained eight MEJA and eight CASJ demonstrators to open either the top or left-side door locks. By training demonstrators to open specific locks at different loci, we can disentangle which social learning mechanism naïve jays may use (main text Table 1). We initially planned to assess evidence for the social learning mechanism of imitation as well by training the demonstrators to use either of two distinct lock opening behaviors. Unfortunately, during social learning trials demonstrators did not consistently use the specific method on which they were trained to open their lock (e.g., a demonstrator trained to swivel B door lock up would swivel the lock up or down during trials). Therefore, we could not test for imitation in this experiment.

Due to the differences in species social systems, we used slightly different methods to isolate demonstrator jays for training. In our CASJ population, aggressive territoriality created a situation where we could train a mate pair as demonstrators in the wild, in the middle of their territory where neighboring jays were unable to observe. However, for MEJA it was impossible to separate two individuals from groupmates for training in the wild. Therefore, we took two MEJA from each of the five flocks into captivity in large aviaries on the campus of the Southwestern Research Station for a maximum of three weeks. We added many tree branches, with and without leaves, to the walls of the aviaries to serve as perches and natural cover. We used tarps on the ceiling and walls in one corner to provide shelter from rain and wind. Jays from the same flock were housed together and jays from different flocks did not have visual access into other aviaries containing jays. The MEJA social system precluded identifying the sex of our subjects because this species is not sexually dimorphic. Captive MEJA were fed a maintenance diet of moistened dog food, pieces of fruit, bird seed, meal worms and wax worms. The maintenance diet was removed during daily training sessions, but jays had access to water at all times. During training we put one of the multi-door foraging apparatuses in the aviary with a preferred food (meal worm, wax worm or peanut) in each compartment. We observed from behind a small one-way mirror as the jay interacted with the log. All other training methods are identical for the two species.

We first determined whether demonstrators could efficiently open the simple unlocked door that we planned to train them on (either the top or left-side door). If not, we used a shaping procedure in which the door was gradually closed until the jays learned to manipulate it to gain access to the food compartment. All other doors were left open and empty to discourage interaction at these loci. Next, we habituated jays to the puzzle box with the focal door closed, and the lock on but not engaged. We then moved the stick so that it was in the way of the door, but not engaged, until jays began to manipulate the stick to access the door. Subsequently, we moved the stick so that it was incrementally more fully in the locked position.

We progressed with the learning trials in each group only after demonstrators were efficiently opening only the trained option on every visit to the log during training sessions on 3 consecutive days, and all naïve jays in the wild were eating comfortably from the compartments of the open puzzle box. Although we specifically trained demonstrators, we used an open diffusion experimental design, so any jay that innovated a solution during social learning trials could serve as a demonstrator.

During each trial, all jays could freely interact with the apparatuses. After jays opened and ate the food item from a locus, the experimenter approached to replace the food, close and lock the door. To prevent the jays from learning about the affordances of each door, the experimenter used their body to block the view of the jays while rebaiting the locus. Although jays may flush while the apparatus is being reset, whistling was almost always successful in bringing the jays back after the experimenter retreated.

S2. Behavioral ethogram and video coding methods

All trials were filmed, and behaviors coded later by individuals naïve to hypotheses according to the ethogram in Table S2.1. Each coder was trained by the first author over two, approximately 1.5-hour sessions. All subsequent, independent coding was double-checked by the first author until each coder had transcribed behavior from at least two 30-minute video clips without a mistake. We used two Canon Powershot digital cameras to simultaneously record jays interacting at each puzzle box with the field of view centered on the puzzle box and 2 m radius of ground around it. Each time a jay interacted with a locus during a trial, we recorded: the time, the individual’s identity, the apparatus (left or right), the door type, whether a food item was successfully obtained, and the identities of all other individual jays in the frame. During trials an experimenter recorded all jays within 10 m of the task that potentially observed each interaction but would not be visible on the video files. Each interaction started when the jay touched the apparatus and ended when the jay moved to a new locus, left the video screen, or succeeded in getting a food item. We then coded that interaction bout as one behavior. For example, if several seconds of attempting at a door resulted in the jay successfully opening it, the whole interaction was coded as “Success” and not as “Attempt”. Additionally, we only coded when individuals observed another jay attempting or succeeding at the apparatus and did not code observations of Scrounge. In our analyses, time to first interaction comprised the first attempt or success (whichever came first) at each locus.

Table S2.1: Ethogram used to code behaviors of jays at the puzzle boxes. Each behavior was also combined with the specific puzzle box (left or right) and specific door (top, left-side, front or right-side) upon which it occurred.

| Behavior | Definition |
| --- | --- |
| Attempt | Touching a closed and locked door with the beak |
| Success | Obtained a peanut after unlocking and opening the door |
| Scrounge | Obtained a peanut after another jay opened the door |
| Observed Attempt | Another jay was within 10m when a group mate touched a door or lock |
| Observed Success | Another jay was within 10m when a group mate opened a door and ate the peanut |

S3. Model specification details

Here we provide a model specification for our baseline Cox Proportional Hazards model:

$${\lambda(t)}_{i,g,a,d}=\lambda0\left( t \right)\times exp\left[ {Group}_{g}+{ID}_{i,g}+{Apparatus}_{g}+{Door}_{d}+\beta1\times{Juvenile}_{i,g}+\beta2\times{Habituation}_{i,g}+\beta3\times{Scrounge\left( t \right)}_{i,g} \right]\times\left[ 1-{Z(t)}_{i,g} \right]$$

For all model parameters and covariates, (t) indicates that the variable changes with time, i indexes individual (1-n_g_), g indexes group (1-10), a indexes apparatus (1-2, representing left or right), and d indexes door type (1-4). The parameter (t)_i,g,a,d_ is the interaction rate for individual i in group g on door type d of apparatus a, and 𝜆0(t) is the underlying learning rate for adults with no prior experience with the puzzle box. *Group*, *ID*, *Apparatus*, and *Door* were normally distributed random effects controlling for non-independence of observations. *Juvenile* takes a value of 1 if the individual is a hatch-year bird, and 0 otherwise. *Habituation* represents the amount of time the individual was able to eat from the unlocked apparatus prior to the experiment and *Scrounge* is the number of times the individual was able to steal food from any compartment without opening the door during the experiment. Finally, we ensured we only modeled the rate of first interaction at each door by including Z, which takes a value of 1 if the individual previously interacted with door type d on apparatus a, and 0 otherwise.

We next tested the effects of personal information on interaction rate by augmenting the baseline model with an additive effect of *Personal*, representing the number of personal successes the individual had at any door prior to interacting with the novel focal door:

$${\lambda(t)}_{i,g,a,d}=\lambda0\left( t \right)\times exp\left[ {Group}_{g}+{ID}_{i,g}+{Apparatus}_{g}+{Door}_{d}+\beta1\times{Juvenile}_{i,g}+\beta2\times{Habituation}_{i,g}+\beta3\times{Scrounge\left( t \right)}_{i,g}+\beta4\times{Personal(t)}_{i,g} \right]\times\left[ 1-{Z(t)}_{i,g} \right]$$

We then tested the effects of four social learning mechanisms on interaction rate using the following model:

$${\lambda\left( t \right)}_{i,g,a,d}=\lambda0\left( t \right)\times exp\left[ {Group}_{g}+{ID}_{i,g}+{Apparatus}_{g}+{Door}_{d}+\beta1\times{Juvenile}_{i,g}+\beta2\times{Habituation}_{i,g}+\beta3\times{Scrounge\left( t \right)}_{i,g}+\beta4\times{Social(t)}_{i,g} \right]\times\left[ 1-{Z\left( t \right)}_{i,g} \right]$$

Here, *Social* represents one of the four social learning mechanisms:

- *SocialFacilitation_i,g_*
- *StimulusEnhancement_i,g,d_*
- *LocalEnhancement_i,g,a_*
- *Emulation_i,g,a,d_*

Finally, we fit models that included additive and interactive effects between *Personal* and each of the four social learning mechanisms (not shown).

S4. Full model results

**Table S4.1**. Estimates and 95% Wald confidence intervals for parameters in all models built to examine the effects of personal and social information on rates of attempting doors in a novel foraging task. Estimates are reported on the scale of the interaction ratio and should be interpreted as the change in the relative interaction rate for each unit increase in the covariate. For example, a value of 2 indicates the relative probability of interacting with a novel door doubles when the covariate increases by 1.

| Species | Model | AIC | Delta AIC | AIC Weight | Juvenile | Habituation | Scrounge | Social | Personal | Social*Personal |
| --- | --- | --- | --- | --- | --- | --- | --- | --- | --- | --- |
|  |  |  |  |  |  |  |  |  |  |  |
| California scrub-jay | |  |  |  |  |  |  |  |  |  |
|  | Personal | 1194.70 | 0.00 | 0.22 | 3.77 (0.78, 18.13) | 1.00 (1.00, 1.00) | 0.91 (0.55, 1.50) | -- | 0.97 (0.91, 1.03) | -- |
|  | SocialFacilitation+Personal | 1196.23 | 1.53 | 0.10 | 3.75 (0.78, 18.06) | 1.00 (1.00, 1.00) | 0.92 (0.55, 1.55) | 0.99 (0.91, 1.08) | 0.97 (0.91, 1.03) | -- |
|  | Baseline | 1196.26 | 1.56 | 0.10 | 3.51 (0.80, 15.31) | 1.00 (1.00, 1.00) | 0.89 (0.55, 1.44) | -- | -- | -- |
|  | StimulusEnhancement+Personal | 1196.50 | 1.80 | 0.09 | 3.76 (0.78, 18.15) | 1.00 (1.00, 1.00) | 0.91 (0.55, 1.50) | 0.99 (0.90, 1.10) | 0.97 (0.91, 1.03) | -- |
|  | LocalEnhancement+Personal | 1196.67 | 1.97 | 0.08 | 3.80 (0.79, 18.19) | 1.00 (1.00, 1.00) | 0.90 (0.54, 1.48) | 1.03 (0.94, 1.11) | 0.97 (0.91, 1.03) | -- |
|  | Emulation+Personal | 1196.68 | 1.98 | 0.08 | 3.77 (0.78, 18.15) | 1.00 (1.00, 1.00) | 0.91 (0.55, 1.49) | 1.01 (0.89, 1.15) | 0.97 (0.91, 1.03) | -- |
|  | SocialFacilitation | 1197.39 | 2.69 | 0.06 | 3.48 (0.79, 15.33) | 1.00 (1.00, 1.00) | 0.92 (0.55, 1.52) | 0.98 (0.91, 1.07) | -- | -- |
|  | Emulation*Personal | 1197.71 | 3.01 | 0.05 | 3.69 (0.77, 17.62) | 1.00 (1.00, 1.00) | 0.91 (0.55, 1.49) | 1.06 (0.92, 1.23) | 0.98 (0.92, 1.05) | 0.98 (0.95, 1.02) |
|  | StimulusEnhancement | 1197.89 | 3.19 | 0.04 | 3.50 (0.80, 15.33) | 1.00 (1.00, 1.00) | 0.89 (0.55, 1.44) | 0.99 (0.90, 1.09) | -- | -- |
|  | StimulusEnhancement*Personal | 1197.96 | 3.26 | 0.04 | 3.70 (0.78, 17.64) | 1.00 (1.00, 1.00) | 0.90 (0.55, 1.48) | 1.02 (0.91, 1.14) | 0.98 (0.92, 1.05) | 0.99 (0.97, 1.01) |
|  | Emulation | 1198.25 | 3.55 | 0.04 | 3.51 (0.81, 15.30) | 1.00 (1.00, 1.00) | 0.89 (0.55, 1.44) | 1.01 (0.89, 1.14) | -- | -- |
|  | LocalEnhancement | 1198.37 | 3.67 | 0.03 | 3.54 (0.82, 15.29) | 1.00 (1.00, 1.00) | 0.88 (0.54, 1.43) | 1.02 (0.94, 1.11) | -- | -- |
|  | SocialFacilitation*Personal | 1198.51 | 3.81 | 0.03 | 3.71 (0.79, 17.47) | 1.00 (1.00, 1.00) | 0.92 (0.55, 1.55) | 1.00 (0.91, 1.10) | 0.98 (0.90, 1.07) | 1.00 (0.99, 1.01) |
|  | LocalEnhancement*Personal | 1198.93 | 4.23 | 0.03 | 3.74 (0.81, 17.34) | 1.00 (1.00, 1.00) | 0.90 (0.55, 1.48) | 1.04 (0.95, 1.15) | 0.98 (0.91, 1.06) | 1.00 (0.99, 1.01) |
|  |  |  |  |  |  |  |  |  |  |  |
| Mexican jay | |  |  |  |  |  |  |  |  |  |
|  | SocialFacilitation+Personal | 2398.78 | 0.00 | 0.39 | 3.01 (1.12, 8.09) | 1.00 (1.00, 1.00) | 1.11 (1.01, 1.22) | 0.95 (0.90, 1.00) | 0.99 (0.95, 1.02) | -- |
|  | SocialFacilitation | 2399.37 | 0.59 | 0.29 | 2.86 (1.10, 7.42) | 1.00 (1.00, 1.00) | 1.11 (1.01, 1.23) | 0.94 (0.90, 0.99) | -- | -- |
|  | SocialFacilitation*Personal | 2400.95 | 2.17 | 0.13 | 3.00 (1.12, 8.03) | 1.00 (1.00, 1.00) | 1.11 (1.01, 1.23) | 0.95 (0.90, 1.00) | 0.99 (0.93, 1.05) | 1.00 (1.00, 1.00) |
|  | LocalEnhancement+Personal | 2402.00 | 3.22 | 0.08 | 2.90 (1.11, 7.62) | 1.00 (1.00, 1.00) | 1.07 (0.99, 1.15) | 0.94 (0.88, 1.00) | 0.98 (0.95, 1.02) | -- |
|  | LocalEnhancement | 2403.56 | 4.77 | 0.04 | 2.72 (1.08, 6.84) | 1.00 (1.00, 1.00) | 1.07 (0.99, 1.15) | 0.94 (0.88, 1.00) | -- | -- |
|  | LocalEnhancement*Personal | 2404.71 | 5.93 | 0.02 | 2.86 (1.10, 7.45) | 1.00 (1.00, 1.00) | 1.07 (0.99, 1.15) | 0.95 (0.88, 1.01) | 0.99 (0.95, 1.04) | 1.00 (0.99, 1.00) |
|  | Personal | 2404.97 | 6.19 | 0.02 | 2.84 (1.10, 7.34) | 1.00 (1.00, 1.00) | 1.02 (0.96, 1.08) | -- | 0.98 (0.95, 1.01) | -- |
|  | StimulusEnhancement+Personal | 2406.76 | 7.98 | 0.01 | 2.85 (1.10, 7.34) | 1.00 (1.00, 1.00) | 1.02 (0.95, 1.10) | 0.99 (0.91, 1.08) | 0.98 (0.95, 1.01) | -- |
|  | Emulation+Personal | 2406.89 | 8.1.0 | 0.01 | 2.84 (1.10, 7.31) | 1.00 (1.00, 1.00) | 1.02 (0.96, 1.09) | 0.98 (0.85, 1.13) | 0.98 (0.95, 1.01) | -- |
|  | StimulusEnhancement*Personal | 2407.84 | 9.06 | 0.00 | 2.89 (1.11, 7.51) | 1.00 (1.00, 1.00) | 1.03 (0.95, 1.10) | 0.98 (0.88, 1.08) | 0.98 (0.94, 1.02) | 1.00 (1.00, 1.01) |
|  | Baseline | 2407.98 | 9.2.0 | 0.00 | 2.61 (1.07, 6.37) | 1.00 (1.00, 1.00) | 1.01 (0.96, 1.07) | -- | -- | -- |
|  | Emulation*Personal | 2408.97 | 10.19 | 0.00 | 2.83 (1.10, 7.29) | 1.00 (1.00, 1.00) | 1.02 (0.96, 1.09) | 0.98 (0.83, 1.16) | 0.98 (0.95, 1.02) | 1.00 (0.99, 1.01) |
|  | StimulusEnhancement | 2409.47 | 10.69 | 0.00 | 2.62 (1.07, 6.42) | 1.00 (1.00, 1.00) | 1.02 (0.95, 1.10) | 0.98 (0.90, 1.07) | -- | -- |
|  | Emulation | 2409.64 | 10.86 | 0.00 | 2.61 (1.07, 6.38) | 1.00 (1.00, 1.00) | 1.02 (0.96, 1.09) | 0.97 (0.85, 1.11) | -- | -- |
|  |  |  |  |  |  |  |  |  |  |  |


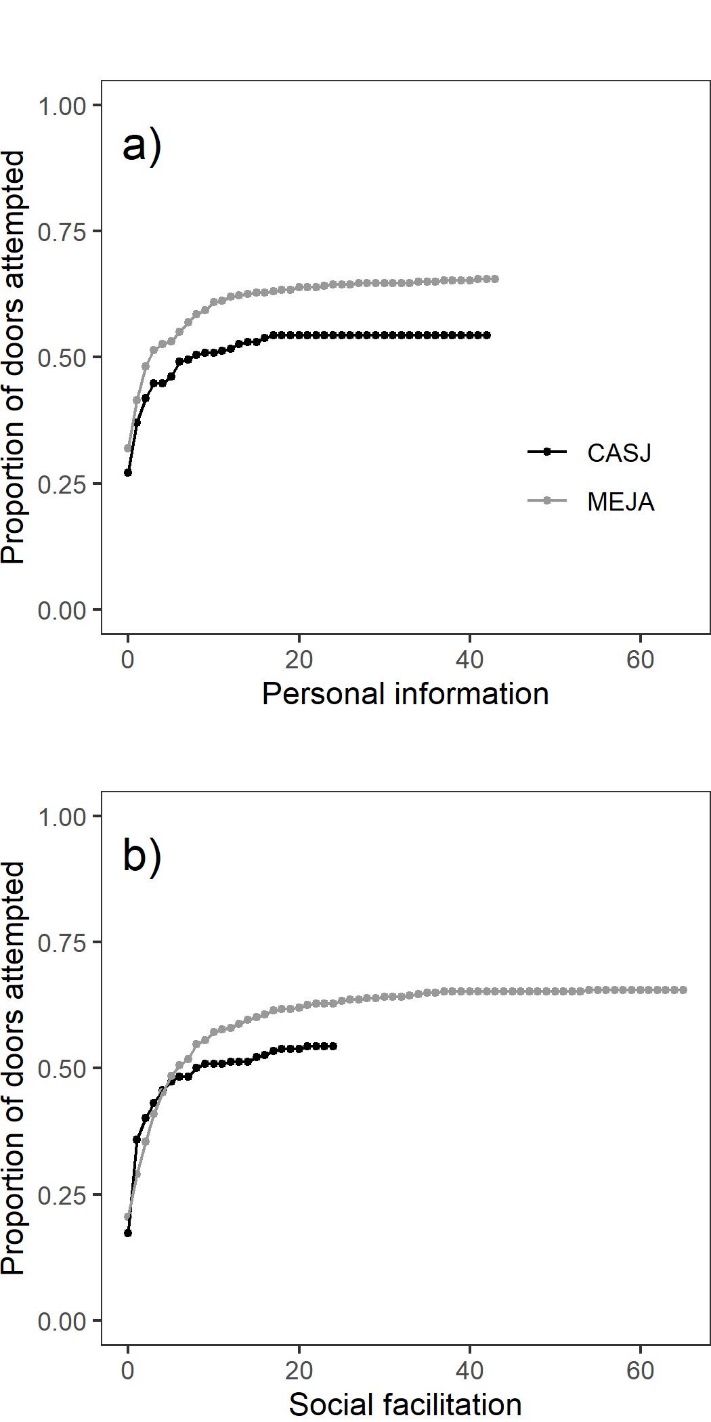


**Figure S4.1:** Our sample of naïve California scrub-jays (CASJ) and Mexican jays (MEJA) that participated in our multi-door foraging task respectively resulted in 232 and 376 potential first attempts with novel doors. The plotted curves illustrate the cumulative proportion of those first attempts that had occurred with a) each new personal success and b) each new observed success for each species. Top models indicated both personal information and social facilitation negatively influenced the rate at which CASJ and MEJA interact with novel doors. Note that all lines asymptote well before 100% of doors are attempted, indicating few novel doors are attempted after about 10 personal or observed successes.
